# Impact of public health expenditure on malnutrition among Peruvians during the period 2010-2020: A panel data analysis

**DOI:** 10.12688/f1000research.153477.2

**Published:** 2024-10-28

**Authors:** Percy Junior Castro Mejía, Rogger Orlando Morán Santamaría, Yefferson Llonto Caicedo, Francisco Eduardo Cúneo Fernández, Nikolays Pedro Lizana Guevara, Milagros Judith Pérez Pérez, Lindon Vela Meléndez

**Affiliations:** 1 Universidad Cesar Vallejo, Avenida Víctor Larco, Trujillo, La Libertad, 1700, Peru; 2 Universidad Nacional Pedro Ruiz Gallo, Juan XXIII 391, Lambayeque, 14013, Peru

**Keywords:** public health expenditure, malnutrition, economy, panel data.

## Abstract

**Background:**

The study analyzes the impact of public health spending on malnutrition among Peruvians, using data from the National Household Survey, the Central Reserve Bank of Peru, the National Institute of Statistics and Informatics and the Ministry of Economy and Finance from 2010. -2020. Previous studies have revealed the existing relationship of health spending with the reduction of malnutrition.

**Methods:**

A quantitative approach is considered, with an explanatory type of research using panel data methodology considering the bidimensionality of the data, which allows quantifying this effect for the Peruvian case using the National Household Survey, data from the Central Reserve Bank of Peru, as well as information from the National Institute of Statistics and Informatics and the Transparency Portal of the Ministry of Economy and Finance in the period 2010-2020.

**Results:**

The results show that public expenditure on health has a negative relationship with malnutrition; the rural sector has a positive relationship with malnutrition given the limitations present for access to adequate food. Similarly, the unemployment rate shows a positive relationship with malnutrition, given that being unemployed leads to a higher cause of malnutrition in the population, and the gross domestic product has a negative relationship with malnutrition, given that greater economic growth produces an impact on reducing malnutrition, with the greatest impact being on the rural population and the gross domestic product.

**Conclusions:**

In the analysis period 2010-2020 in Peru, based on the panel data analysis, the impact of public health expenditure on reducing malnutrition is observed in 10 departments, achieving a reduction in malnutrition; while in 14 departments, this indicator has not been reduced.

## Introduction

The fight against hunger is considered a persistent problem for various countries around the world. According to the United Nations Food and Agriculture Organization (2021) even before the coronavirus pandemic, it was projected that the sustainable development goal of ending hunger and malnutrition by 2030 would not be achieved ( Bhuyan et al., 2020; Ritchie et al., 2018; Madsen et al., 2022; Mottaleb et al., 2021; Sridhar et al., 2023).

Casado et al. (2021) projected that between 2019 and 2020, nearly 657 million people, representing 8% of the world’s population, would be undernourished by 2030. This led to an increase in the prevalence of undernourishment from 8.4% to 9.9%, primarily concentrated in the regions of Asia (418 million), Africa (282 million), and Latin America and the Caribbean (14 million) ( United Nations Food and Agriculture Organization, 2021; Cambra-Fierro et al., 2022; Liu et al., 2023; Vollset et al., 2024).

The average public health expenditure in Latin America and the Caribbean is 3.8%, showing an increasingly unequal situation. Countries like Paraguay, Costa Rica, Ecuador, and Nicaragua allocate over 9% of their gross domestic product (GDP) to health, while countries like Peru, Jamaica, and Guyana do not exceed 6% of their GDP ( Haldane et al., 2022; Pan American Health Organization, 2018).

In Peru, the problem is no different. The Pan American Health Organization (2018) states that despite favorable economic growth conditions in recent decades, this has not been reflected in higher health expenditure, barely surpassing 3% of GDP in the last ten years. In an optimistic scenario, it would only grow by one percentage point in the next seven years ( Vázquez-Rowe & Gandolfi, 2020).

The National Institute of Statistics and Informatics (2020) analysis revealed that the Peruvian population had a food intake below the required minimum, with a national caloric deficit incidence of 26.8% and a growing trend during the 2016-2019 period, causing malnutrition issues. Vázquez-Rowe & Gandolfi (2020) consider that Peruvian nutrition is subject to an average monthly income of 1,400 soles, which is below a healthy basic basket of 1,515 soles for a household of four.

In urban areas, the daily diet consists of 1,161 grams of food products, resulting in a 22% caloric deficit among the urban population. In rural areas, the daily diet is 938 grams, with a 40.2% caloric deficit, evidencing inadequate nutritional intake due to low incomes that do not allow for the purchase of a basic food basket, with severe consequences in the fight against poverty and malnutrition ( Velásquez et al., 2021).

Calva & Ruiz (2021) state that there is still scant evidence of the relationship between health expenditure and malnutrition, with malnutrition being one of the social problems that directly impacts a country’s economic development ( Caballero & Sánchez, 2021; Leung & Wolfson, 2019; Mogues & Billings, 2019).

For this reason, the research is justified by its practical contribution in revealing the relationship between health expenditure and malnutrition during the analysis period 2010-2020, considering the critical aspect Peru faces in its latest figures of increasing poverty and inequality, which indicate failure to meet the sustainable development goal of ending poverty and inequality.

## Literature review

At the international level ( Katoch, 2022), following a systematic review of the literature, he considers that the most relevant factors associated with child undernutrition are the mother’s education, family income, the mother’s nutritional status, the child’s age, the availability of sanitary facilities in the home, the size of the family, the birth order in the family and the child’s birth weight.

Importantly, Katoch (2024) evidence that malnutrition is associated with energy poverty, which has negative effects on health and education, whose efforts imply that economic conditions are determinant for reaching optimal levels of access to energy services and achieving quality of life in studies conducted in Asia, Europe, Africa and North America.

Katoch et al. (2024) starts from the objective of assessing the achievements of Sustainable Development Goal 2, where it is evident that after having carried out the analysis of the challenges regarding hunger, the most relevant energy measures were adopted to address the persistent challenges of malnutrition and hunger, leading to identify that the highest percentage of states and territories are classified as aspiring and good performers.

Bernet et al. (2018) considers that the effectiveness of public health expenditure shows the challenges of aggregate public health expenditures and the problem of endogeneity and serial correlation between expenditures and outcomes; finding the inverse association between public health spending and the infant mortality rate.

Similarly, Calva & Ruiz (2021) showed that in countries in Latin America and Sub-Saharan Africa, public spending on health has a negative relationship with malnutrition; considering that in the rural sector, given the characteristics they face, they have a greater impact on malnutrition, showing a positive relationship; while unemployment has a positive relationship with malnutrition, just as inflation has a positive relationship with malnutrition.

Edney et al. (2018) in their study carried out in Australia shows that a 1% increase in public health spending is associated with a 2.2% reduction in the number of years lost, showing that the marginal effects of public health spending are more effective in those areas where there are weak health systems.

On the other hand, Antelo et al. (2017) analyzes unemployment and its relationship with food spending in Spanish households, showing that unemployment has a negative impact on malnutrition, the greatest intensity of which is seen in economic crises; socioeconomic households being those that have the greatest impact on lower food consumption. Hence Bernet et al. (2018) points out that high levels of unemployment and poverty are the main causes of food insecurity, having an environment of complex causality structure in the design of policies for food assistance.

Guo et al. (2019) points out that education also has an impact on reducing malnutrition, given that when the rural population migrates to urban areas they have a greater probability of better education, since Deller et al. (2019) points out that the rural population has important impact results in terms of education compared to the urban population.

Fallah et al. (2021) points out that the double burden of malnutrition has become a growing health problem, given the dynamics of energy imbalance that causes public health interventions to be oriented towards a reduction in energy imbalance, mainly by slowing down the prevalence of obesity and overweight. However ( Velásquez et al., 2021) considers that in the Peruvian case, the diet in six regions shows a high consumption of carbohydrates and an unbalanced diet that leads to a critical state of nutrition because low income does not It allows them to purchase the basic food basket, showing greater growth in morbidity and mortality.

For Huaripuma (2022); Vera (2019) and Guzmán (2021) demonstrate in their research in Peru the indirect relationship between public spending on health and the decrease in the percentage of chronic malnutrition in children under five years of age. The challenge being to improve the indicators in terms of indicators of prenatal health, child health or chronic malnutrition given that the three impact evaluations of social programs using the family visiting service.

## Methods

From the review of the literature it is observed that the importance of public health spending in reducing malnutrition has been methodologically addressed by the vast majority of studies using the quantitative method and based on regressions of socioeconomic and macroeconomic variables ( Bernet et al., 2018; Calva & Ruiz, 2021; Edney et al., 2018; Antelo et al., 2017; Guo et al., 2019; Fallah et al., 2021; Huaripuma, 2022; Vera, 2019; Guzmán, 2021).

### Methodological design

The methodological approach considers the traditional quantitative approach and correlational type of research, given that it analyzes the impact of public health spending on malnutrition among Peruvians, using data from the National Household Survey, the Central Reserve Bank of Peru, the National Institute of Statistics and Informatics and the Ministry of Economy and Finance from 2010-2020.

### Procedure

The data comprises a total of 2,112 observations covering the 24 departments of Peru during the period 2010-2020. The dependent variable is malnutrition, measured by caloric deficit, while the independent variable is public health expenditure, incorporating control variables such as inflation, rural population, unemployment, education, exports, and gross domestic product. The analysis was conducted over the 2010-2020 period based on data availability, considering inclusion and exclusion criteria.

The inclusion criteria were Peruvian households experiencing a caloric deficit. We defined caloric deficit according to the methodology used by Herrera (2002), which establishes a caloric norm for Peru of 2,318 calories per capita per day. This caloric norm is derived from data from the National Household Survey conducted by the National Institute of Statistics and Informatics between the years 2010 and 2020.

While the exclusion criteria is peruvian households not experiencing a caloric deficit according to the methodology used by Herrera (2002) which defines the caloric norm for Peru as a total of 2,318 calories per capita per day, based on data from the National Household Survey by the National Institute of Statistics and Informatics for the years 2010 to 2020.

### Sample

The sample size consists of a total of 2112 observations covering the 24 departments of Peru during the period 2010-2020, considering that for quantitative research according to Hernández & Mendoza (2018) the minimum sample size for causal or comparative analysis is 64 data, which implies that the sample is non-probabilistic, given the careful and controlled choice of data that have fed the variables previously in the problem statement.

The sampling method is non-probabilistic as it addresses the entirety of the data, totaling 2,112 observations, involving departmental analysis for the period 2010-2020.

The unit of analysis comprises each department, totaling 24 departments in Peru.

Document analysis was used as the technique for measuring the variables, and the observation guide was employed as the instrument. This tool is used to systematically and structurally collect data during an observation or study.

Data were obtained from the microdata database of the National Household Survey for the years 2010 to 2020. The data were extracted from module 01 and 02 (Household Member Characteristics), module 03 (Education), module 04 (Health), module 05 (Employment and Income), module 07 (Food and Beverage Expenditures), and module 08 (Charitable Institutions) published on the website of the National Institute of Statistics and Informatics. This annual survey allows for the tracking of indicators on living conditions and is conducted nationwide across the 24 departments of the country.

Additionally, data on the percentage variation of the price index by department for the period 2010-2020 and the gross value added by department at constant prices from 2007, compiled by the National Institute of Statistics and Informatics, were used and published in its economic statistics section.

For the collection of public health expenditure data, departmental data reflecting the amount spent in millions of soles were obtained from the Economic Transparency Portal of the Ministry of Economy and Finance for the period 2010-2020.

Finally, the database of the value of all goods and other market services exported to the rest of the world by department, measured in FOB value in millions of dollars, from the Central Reserve Bank of Peru for the period 2010-2020 was used. This data reflects the registration of sales abroad of goods and services by a resident company in Peru, including both traditional and non-traditional products.

### Data analysis method

The estimation of malnutrition is based on the estimation of the caloric deficit and the basal metabolic rate using the methodology by Herrera (2002) which defines the caloric norm for Peru as a total of 2,318 calories per capita per day. This is based on the data from the National Institute of Statistics and Informatics, considering the average weight estimates by sex and age, classified for children under 10 years and those over 10 years by FAO ( Swindale & Ohri, 2004).

For calculating the basal metabolic rate (BMR) for those over 10 years old, corrections were made based on estimates for Latin American countries (Bengoa et al., as cited in Herrera, 2002). Two options were considered: moderate activity for those over 10 years old in urban areas and intense activity for those over 10 years old in rural areas.

From the data obtained, which include time series and cross-sectional data, which allows the analysis of multiple observations over time for the same sample unit relevant to each department of Peru analyzed in the period 2010-2020, the econometric model called panel data is used, since it allows obtaining the largest amount of information and at the same time controlling the unobservable variability that is constant over time but may differ between subjects, reducing bias in the results ( Wooldridge, 2010).

The panel data model helps to analyze changes over time and to identify causal relationships between variables that may vary between periods and reduce collinearity for better accuracy of estimates, allowing to increase the efficiency and power of estimates, by obtaining more robust and reliable results ( Wooldridge, 2010).

On the other hand, the Generalized Least Squares (GLS) model is used since it becomes a fundamental tool when working with structural equations that present problems of heteroscedasticity and autocorrelation ( Wooldridge, 2010).

By applying the GLS model, it is possible to obtain regression coefficients that more accurately reflect the relationship between variables, even when the data present variability and dependence in the errors. This is especially important in structural equations, where the complexity of the relationships between variables can make problems of heteroscedasticity and autocorrelation more common and, therefore, the use of GLS is essential to guarantee the validity and precision of the results obtained ( Wooldridge, 2010).

The panel data model, based on Mushkin’s theory, will be used for the estimation, showing the relationship between malnutrition behavior explained by public health expenditure ( Calva & Ruiz, 2021). The model utilizes two equations.

Through this model, the impact of public health expenditure on the malnutrition of Peruvians is quantified. Malnutrition, measured by caloric deficit, is the dependent variable, while public health expenditure is the independent variable. Control variables include inflation, rural population, unemployment, education, exports, and gross domestic product.

To ensure consistent estimations and an adequate analysis, variables such as public health expenditure, rural population, education, exports, and gross domestic product have been transformed into logarithms. Additionally, to estimate the regression with control variables, the structural equations of the variables under analysis are used ( Wooldridge, 2010).

Equation 1 establishes the relationship between the dependent variable malnutrition and public health expenditure by department =1,2, …, 24i=1,2, …, 24 in the year t=2010−2020 t=2010−2020.

$${\text{Desnt}}_{i,t}=\mathrm{Bo}+B_{1}\;\left({\text{gsalud}}_{i,t}\right)+e_{i,t}$$ (1)

On the other hand, the incorporation of control variables for the robustness of the model includes variables such as inflation, rural population, unemployment, education, exports, and gross domestic product, which are reflected in the following equation:

$${\text{Desnt}}_{i,t}=\mathrm{Bo}+B_{1}\;\left({\text{gsalud}}_{i,t}\;\right)+B_{2}\;\left({\text{infl}}_{i,t}\;\right)+B_{3}\;\left({\text{prural}}_{i,t}\;\right)+B_{4}\;\left({\text{desem}}_{i,t}\;\right)+B_{5}\;\left({\text{educ}}_{i,t}\right)+B_{6}\;\left({\text{export}}_{i,t}\right)+B_{7}\;\left({\mathrm{pbi}}_{i,t}\right)+e_{i,t}$$ (2)

In Equation 2, structural equations will be used to evaluate the interdependencies, expressing the mentioned interrelationship in four equations:

$${\text{Gsalud}}_{i,t}=\mathrm{Bo}+B_{1}\;\left({\text{Desnt}}_{i,t}\right)+B_{4}\;\left({\text{desem}}_{i,t}\right)+e_{i,t}$$ (3)

$${\text{Prural}}_{i,t}=\mathrm{Bo}+B_{1}\;\left({\text{educ}}_{i,t}\right)+e_{i,t}.$$ (4)

$${\text{Desem}}_{i,t}=\mathrm{Bo}+B_{1}\;\left({\text{Desnt}}_{i,t}\;\right)+B_{2}\;\left({\text{infl}}_{i,t}\right)+B_{5}\;\left({\text{educ}}_{i,t}\right)+e_{i,t}$$ (5)

$${\mathrm{Pbi}}_{i,t}=\mathrm{Bo}+B_{1}\;\left({\text{export}}_{i,t}\;\right)+B_{2}\;\left({\text{infl}}_{i,t}\right)+B_{3}\;\left({\text{desem}}_{i,t}\right)+e_{i,t}$$ (6)

Given the presence of heteroscedasticity and autocorrelation in the structural equations, confirmed by Breusch and Pagan (1979) tests for heteroscedasticity and Wooldridge (2010) tests for autocorrelation, a Generalised Least Squares (GLS) model was employed. The GLS estimator accounts for these violations of classical linear regression assumptions, providing more efficient and unbiased estimates. Additionally, a Hausman (1978) specification test was conducted to determine the appropriateness of fixed or random effects.

For the estimation of the relevant control variables for the study according to the objective of the research that seeks to quantify the impact of public health spending on malnutrition of Peruvians during the years 2010-2020, the theoretical review and availability of the data described above have been considered, taking as a basis the theory of ( Mushkin, 1962) where it explains that the behavior of malnutrition is explained by public spending on health and the control variables considered by ( Calva & Ruiz, 2021) that includes inflation, rural population, unemployment, education, exports and gross domestic product in order to reduce the bias of the estimates; applying said model with the data of Peru in the period 2010-2020.

Panel data models are based on several assumptions that allow estimates to be consistent, efficient and unbiased, as detailed below ( Beltrán & Castro, 2010).

• No perfect multicollinearity: It is assumed that there is no exact linear relationship between the independent variables. In other words, no explanatory variable should be an exact linear combination of the others.
• Homoscedasticity and No autocorrelation of errors: In the case of random effects models, it is assumed that the errors have a constant variance and are not correlated over time, considering that Generalized Least Squares was used to correct the problem of heteroscedasticity and autocorrelation.
• No correlation between individual effects and independent variables: In random effects models, it is assumed that individual effects (the variables) are not correlated with the explanatory variables of the model; for our research, fixed effects were used.
• Strict exogeneity: This assumption implies that the errors are not correlated with the independent variables of the model in all time periods for each individual.
• No serial correlation: For fixed effects models, it is assumed that the errors are not correlated over time.

The variables used for the model estimation are detailed in Table 1.

**Table 1.** Variables used in the model.

Variable	Description	Source
Malnutrition (desn i,t)	The population by department located below the minimum level of caloric requirements, expressed as the percentage of the population by region whose intake of safe and nutritious food is insufficient to meet their minimum energy needs	National Household Survey 2010-2020
Public Health Expenditure (gsalud i,t)	Level of health expenditure, expressed by the amount accrued by regional governments in the health function, in thousands of soles	Economic Transparency Portal, Ministry of Economy and Finance 2010-2020
Rural Population (prural i,t)	Population by department in rural areas, calculated as the difference between the total population and the urban population	National Household Survey 2010-2020
Unemployment (desem i,t)	Percentage of the active population by region that is unemployed but seeking work and available for work	National Household Survey 2010-2020
Inflation (inf i,t)	Percentage variation of the price index by department	National Institute of Statistics and Informatics
Education (educ i,t)	Average years of education by department	National Household Survey 2010-2020
Exports (export i,t)	Value of all goods and other market services exported to the rest of the world by department, in thousands of soles	Central Reserve Bank of Peru 2010-2020
Gross Domestic Product (y i,t)	Gross Value Added by department, at constant 2007 prices, in thousands of soles	National Institute of Statistics and Informatics

*Note.* Variables considered in the estimation of the proposed equations.

### Data processing

For the estimation of the undernutrition variable, the National Household Survey database for the period 2010-2020 was considered. Eviews 12 is used for the estimation of the panel data model, considering the control variables that are subjected to the proposed equations to analyze the interrelationship between the variables. This allows for both descriptive statistical treatment and the estimation of the proposed panel data model and the Generalized Least Squares (GLS) model. An academic license for the use of Eviews 12 software is available, registered under the name Lindon Vela Meléndez. The license details are as follows: Serial number: Q1208886 - D49010AF - 9D854485. The software can be downloaded from the following link: http://www.eviews.com/download/student12.

Autocorrelation detection was performed using the Lagrange multiplier tests of Breusch & Pagan (1979) and Wooldridge (2010). In addition, the Hausman (1978) test was used to choose between a fixed or random effects model.

We assessed the presence of autocorrelation in the model residuals using two statistical tests. First, the Breusch and Pagan (1979) Lagrange multiplier test was applied, which examines the null hypothesis of no autocorrelation against the alternative of autocorrelation up to a specified order. Second, the Wooldridge (2010) test, designed specifically to detect first-order autocorrelation in panel data, was used.

To determine the appropriate specification of the model, we perform a Hausman (1978) specification test. This test compares fixed and random effects estimators under the null hypothesis that individual specific effects are uncorrelated with the explanatory variables.

### Ethical considerations

Data for this study were obtained from the National Household Survey (ENAHO) conducted by the National Institute of Statistics and Informatics (INEI). The ENAHO, as a publicly available and freely accessible dataset on the INEI website, adheres to rigorous ethical standards to protect the confidentiality of participants.

Furthermore, ENAHO data by INEI are anonymised before being made publicly available. This ensures that individual responses cannot be linked to specific participants.

Although we cannot provide specific details about the consent process for each individual participant in the ENAHO survey, INEI, as a reputable government institution, is committed to ethical research practices and to obtaining informed consent.

In the ethical use of public data obtained by the INEI in the ENAHO, we adhere to the principles of intellectual honesty, truthfulness, transparency, human integrity, respect for intellectual property, justice and responsibility, the study aims to comply with the “Code of Ethics in Research of the Universidad César Vallejo, version 01; by University Council Resolution N° 0340-2021-UCV”.

We have used the data for research purposes only, as permitted by INEI’s terms of use. We have not attempted to re-identify any individual, and our analysis does not include any personally identifiable information.

By using publicly available anonymised data and adhering to ethical principles in our research, we aim to minimise any potential ethical concerns while leveraging valuable information for the benefit of public health research.

## Results

The results of the estimated regressions, considering the Wooldridge test (2010), indicate the presence of autocorrelation in the various panels. Additionally, the Breusch and Pagan (1979) Lagrange Multiplier test shows that the estimates presented heteroscedasticity issues. These issues have been corrected using Generalized Least Squares (GLS) to address the problems of heteroscedasticity and autocorrelation.

### Regression of malnutrition with control variables

Table 2 shows the results of the panel data estimation, where it is observed that public health expenditure, rural population, unemployment, and gross domestic product are statistically significant at the 5% level, considering Equation 2.

**Table 2.** Regression of malnutrition with control variables.

Explanatory variable	Levels	Fixed effects	Random effects
Constant	3.0357	107.5500	48.1448
	*(0.8059)	*(0.0000)	*(0.0432)
Log_salud	-2.7035	-2.5631	-2.5375
	*(0.0000)	*(0.0000)	*(0.0000)
Log_rural	11.4642	4.5941	5.3864
	*(0.0000)	*(0.017)	*(0.0015)
Desem	6.2010	2.2766	2.3181
	*(0.0000)	*(0.0000)	*(0.0007)
Log_y	-1.2916	-4.2847	-0.9685
	*(0.0317)	*(0.036)	*(0.4510)
R-squared	0.332521	0.856703	0.390645
Test Hausman		0.0000	0.0000
Test Breusch-Pagan	0.0000	0.0000	0.0000
Durbin Watson stat	0.623864	1.28741	1.15187
Fixed effects		Yes	Yes
Dynamic Effects		No	No
Observations	264	264	264

*Note. Abbreviations used:* “Log_salud” = Logarithm Public Health Expenditure; “Log_rural” = Logarithm Rural Population; “Desem” = Unemployment; Log_y = Logarithm Gross Domestic Product. Due to the type of information collected, the panel data model is used.

* Significance p<0.05.

It is observed that for each 1% increase in public health expenditure, malnutrition decreases by 2.6%. Conversely, a 1% increase in the rural population leads to a 4.6% increase in malnutrition. Additionally, a 1% increase in the unemployment rate results in a 2.3% increase in malnutrition, while a 1% increase in gross domestic product leads to a 4.3% decrease in malnutrition. The variables with the most significant impact during the analysis period 2010-2020 are the rural population and the gross domestic product.

### Regression of public health expenditure with malnutrition and unemployment

Table 3 shows the analysis of public health expenditure considering structural Equation 3. The results indicate that chronic malnutrition and unemployment are significant at the 5% confidence level. It explains that malnutrition has a negative effect on public health expenditure due to the limited importance given to public malnutrition policies, with public budgets being prioritized for other sectors.

**Table 3.** Regression of public health expenditure with malnutrition and unemployment.

Explanatory variable	Levels	Fixed effects	Random effects
Constant	20.3901	22.7669	20.7418
	*(0.0000)	*(0.0000)	*(0.0000)
Desn	-0.0602	-0.1598	-0.0751
	*(0.0000)	*(0.0000)	*(0.0000)
Desem	0.1136	0.3977	0.1575
	*(0.3142)	*(0.0114)	*(0.2568)
R-squared	0.1395	0.447182	0.178649
Test Hausman		0.0000	0.0000
Test Breusch-Pagan	0.0000	0.0000	0.0000
Durbin Watson stat	1.842586	1.80502	1.82413
Fixed effects		Yes	Yes
Dynamic effects		No	No
Observations	264	264	264

*Note. Abbreviations used:* “Desn” = Malnutrition; “Desem” = Unemployment. Due to the type of information collected, the panel data model is used.

* Significance p<0.05.

It was observed that for each 1% increase in the malnutrition rate, public health expenditure decreases by 0.16%. Meanwhile, a 1% increase in the unemployment rate leads to a 0.40% increase in malnutrition.

### Regression of rural population with education

Table 4 shows the analysis of the rural population considering structural Equation 4. The results indicate that education is significant at the 5% confidence level, explaining that education has a negative effect on the rural population. This is mainly due to the trend that as the human capital of the population increases, there is a migration from rural to urban areas, moving to central zones to improve their income levels.

**Table 4.** Regression of rural population with education.

Explanatory variable	Levels	Fixed effects	Ramdom effects
Constant	13.0294	10.3153	10.9851
	*(0.0000)	*(0.0000)	*(0.0000)
Log_educ	-2.8455	-1.4636	-1.8046
	*(0.0000)	*(0.0000)	*(0.0000)
R-squared	0.569582	0.952405	0.129147
Test Hausman		0.0000	0.0000
Test Breusch-Pagan	0.0000	0.0000	0.0000
Durbin Watson stat	0.214215	1.31476	1.09918
Fixed effects		Yes	Yes
Dynamic effects		No	No
Observations	264	264	264

*Note. Abbreviations used:* “Log_educ” = Logarithm Education. Due to the type of information collected, the panel data model is used.

* Significance p<0.05.

It was observed that for each 1% increase in the average years of education, the malnutrition rate decreases by 1.46%.

### Regression of unemployment with education, malnutrition, and inflation

Table 5 shows the analysis of unemployment considering structural Equation 5. The results indicate that chronic malnutrition, inflation, and education are significant at the 5% confidence level. It explains that malnutrition has a positive effect on unemployment, as do education and inflation. This shows that high levels of malnutrition lead to high unemployment rates, indicating that nutritional imbalance causes problems in performing physical or intellectual labor, affecting worker productivity.

**Table 5.** Regression of unemployment with education, malnutrition, and inflation.

Explanatory variable	Levels	Fixed effects	Random effects
Constant	-6.975429	-1.781961	-5.6962
	*(0.0000)	*(0.0188)	*(0.0000)
Log_educ	4.306179	1.800031	3.7822
	*(0.0000)	*(0.0148)	*(0.0000)
Desn	0.019167	0.010627	0.0108
	*(0.0000)	*(0.0025)	*(0.0179)
Inf	0.014043	0.009445	0.0141
	*(0.0434)	*(0.0390)	*(0.0200)
R-squared	0.470294	0.696024	0.205113
Test Hausman		0.0000	0.027500
Test Breusch-Pagan	0.0000	0.0000	0.0000
Durbin Watson stat	0.983788	0.86534	1.245992
Fixed effects		Si	Si
Dynamic effects		No	No
Observations	264	264	264

*Note. Abbreviations used:* “Log_educ” = Logarithm Education; “Desem” = Unemployment; “Inf” = Inflation. Due to the type of information collected, the panel data model is used.

* Significance p<0.05.

Additionally, the positive relationship between education and unemployment is explained by the excess accumulation of human capital in Peru, which exceeds the available job opportunities due to limited positions. Regarding inflation, the positive relationship with unemployment contradicts the Phillips Curve theory (1958) which suggests an inverse relationship between inflation and unemployment. According to Campoverde et al. (2016) this positive relationship depends on the economic context of each country. In Peru, the loss of purchasing power and delayed investment due to price fluctuations result in a decline in business confidence, creating an uncertain climate that impacts higher unemployment rates.

It was observed that for each 1% increase in the malnutrition rate, unemployment increases by 0.01%. Additionally, a 1% increase in the average years of education leads to a 1.80% increase in unemployment, and a 1% increase in inflation causes unemployment to rise by 0.01%.

### Regression of gross domestic product with exports, unemployment, and inflation

Table 6 shows the analysis of gross domestic product (GDP) considering structural Equation 6. The results indicate that exports, unemployment, and inflation are significant at the 5% confidence level. The findings explain that exports have had a positive effect on GDP during the analyzed period, as higher exports result in a positive balance of payments, impacting foreign exchange and terms of trade, thereby fostering GDP growth. Conversely, unemployment shows a negative relationship with GDP due to the still high unemployment rates in Peru, which negatively affect economic growth by reducing private investment and capital attraction. Inflation also shows a negative relationship with GDP, as the inflationary spiral leads to a loss of purchasing power, consequently affecting economic growth. This is consistent with Fernando (2016), who evidenced the negative relationship between inflation and economic growth for Honduras, and Uribe (2006), who noted that demand shocks negatively affect GDP and prices in Bolivia, while supply shocks have had a positive long-term impact on GDP and a decline in price levels.

**Table 6.** Regression of gross domestic product.

Explanatory variable	Levels	Fixed effects	Random effects
Constant	10.0677	14.7209	13.7375
	*(0.0000)	*(0.0000)	*(0.0000)
Log_export	0.4132	0.0957	0.1659
	*(0.0000)	*(0.0000)	*(0.0000)
Desem	0.0491	-0.0435	-0.0468
	*(0.2850)	*(0.0000)	*(0.0132)
Inf	-0.0065	-0.0032	-0.0024
	*(0.3115)	*(0.0000)	*(0.0939)
R-squared	0.627458	0.98757	0.319472
Test Hausman		0.0000	0.0000
Test Breusch-Pagan	0.0000	0.0000	0.0000
Durbin Watson stat	0.0874	0.52895	0.39419
Fixed effects		Si	Si
Dynamic effects		No	No
Observations	264	264	264

*Note. Abbreviations used:* “Log_export” = Logarithm Exports; “Desem” = Unemployment; “Inf” = Inflation. Due to the type of information collected, the panel data model is used.

** Significance p<0.05.

It was detailed that for each 1% increase in the value of exports, GDP increases by 9.5%. Meanwhile, for each 1% increase in the unemployment rate, GDP decreases by 4.3%, and a 1% increase in inflation would cause GDP to decrease by 0.32%.

### Effect of public health expenditure on malnutrition by department

**Figure 1.**
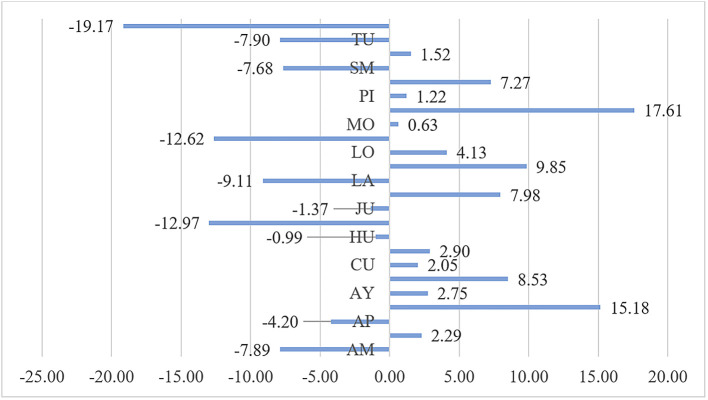
Impact of Public Health Expenditure on Malnutrition by Department. *Note.* The abbreviations of the regions of Peru are AM (Amazonas), AN (Ancash), AP (Apurímac), AR (Arequipa), AY (Ayacucho), CA (Cajamarca), CU (Cusco), HU (Huánuco), HV (Huancavelica), IC (Ica), JU (Junín), LA (Lambayeque), LL (La Libertad), LI (Lima), LO (Loreto), MD (Madre de Dios), MO (Moquegua), PA (Pasco), PI (Piura), PU (Puno), SM (San Martín), TA (Tacna), TU (Tumbes), and UC (Ucayali).

In the analysis of the impact of public health expenditure on the reduction of malnutrition by department shown in Figure 1, it is observed that the greatest impact is in the Ucayali department, which reduced the malnutrition rate by 19.17%, followed by Madre de Dios with a reduction of 12.62%, and Ica with a reduction of 12.97%. Conversely, in Pasco, public health expenditure led to an increase in malnutrition by 17.61%, in Arequipa by 15.18%, in Lima by 9.85%, and in Cajamarca by 8.53%. This variability in the impact of public health expenditure on malnutrition during the period 2010-2020 indicates differing efficiencies and effectiveness of public health spending across departments. In 14 departments, including Pasco, Arequipa, Cajamarca, La Libertad, and Lima, public health expenditure did not achieve a reduction in the malnutrition indicator, with the population not meeting the minimum required caloric intake during the period 2010-2020.

**Figure 2.**
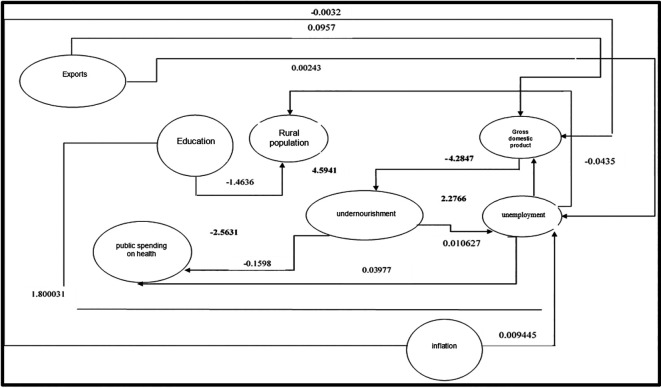
Dynamics of the identified structural equations in the departments of Peru. *Note.* Result of the interactions of regressions in the identified structural equations in the departments of Peru

Figure 2 shows the interrelationship of the variables in the proposed structural equations, where it has been evidenced that public health expenditure has not had the expected impact, as malnutrition levels have increased in Peru. The rural sector shows the most significant relevance with malnutrition due to limitations in accessing adequate food because of low incomes. Thus, the unemployment rate has a positive relationship with malnutrition, demonstrating that the unemployed population would have lower adequate calorie consumption, highlighting a positive association with malnutrition. Meanwhile, the sustained growth of gross domestic product (GDP) produces a negative effect in reducing malnutrition, with the most significant impact being related to the rural population and GDP.

Additionally, it is observed that exports have positively influenced GDP, being considered a pillar of the Peruvian economy’s growth through the diversification of the productive matrix, which leads to a negative relationship with unemployment. Inflation, on the other hand, shows a negative relationship with GDP, as the inflationary spiral causes a loss of purchasing power, thereby affecting economic growth.

## Discussion

The analysis of the impact of public health expenditure on the malnutrition of Peruvians during the years 2010-2020 shows a negative effect of public health expenditure on malnutrition. These results align with Huaripuma (2022), Vera (2019), and Guzmán (2021), who detailed in their research in Peru the indirect relationship between public health expenditure and the reduction of the chronic malnutrition percentage in children under five years old.

It is essential to highlight the interrelation with other variables observed in the proposed structural equations. Literature has evidenced that exports have had a positive effect on GDP during the analyzed period. Higher exports result in a positive balance of payments, impacting foreign exchange and terms of trade, thereby fostering GDP growth. In contrast, unemployment shows a negative relationship with GDP due to the still high unemployment rates in Peru, which negatively affect economic growth by reducing private investment and capital attraction. Inflation also shows a negative relationship with GDP, as the inflationary spiral leads to a loss of purchasing power, consequently affecting economic growth. This is supported by Bhuyan et al. (2020) who found that despite high economic growth in India, the country still faces the global problem of nutritional deficit, with the poor and very poor facing food insecurity alongside the middle class, causing issues among disadvantaged groups.

However, Bernet et al. (2018) consider that the effectiveness of public health expenditure shows the challenges of aggregated public health spending, the problem of endogeneity, and the serial correlation between expenditures and outcomes, finding an inverse association between public health expenditure and the infant mortality rate. Similarly, Biadgilign et al. (2019) evidence that child malnutrition remains a significant problem in low- and middle-income countries, with adequate governance, urbanization, and public health expenditure having effects on child malnutrition.

Peru is considered an upper-middle-income country that has been implementing public policies to reduce poverty through various social programs. According to Brar et al. (2020) a notable reduction in malnutrition has been observed in the Central and Western regions, involving multiple factors such as socioeconomic indicators, reductions in inequalities, and greater access to health services. The latter is a key element in reducing health, water, and sanitation gaps, resulting from better economic conditions due to Peru’s economic growth exceeding 5% of GDP. This regional analysis has allowed for greater public resources via public investment, reducing basic infrastructure gaps and contributing to the fight against malnutrition, corroborating the importance of public expenditure in various basic sectors.

However, Peru faces a double burden of malnutrition, as stated by Quinteros et al. (2024) where not only prioritizing policies on malnutrition due to historically high levels is necessary, but also considering impacts related to intrafamily distribution and food quality used in regional programs like Qaliwarma. Another state program in Peru’s fight against poverty is Juntos, but in terms of cost-effectiveness, it still faces regional challenges, as indicated by Brar et al. (2020). Addressing the underlying causes of household targeting for active policy implementation requires leadership and effectiveness from policymakers and program leaders against malnutrition in the context of a politically uncertain agenda in Peru. This leads to an increased gap between the poor and the rich, as well as between rural and urban areas, and highlights the need for basic health and sanitation infrastructure to support a multidimensional approach beyond just conditional cash transfers.

Thus, the institutional capacity to ensure a common good for the population, such as food and nutritional security, remains critical, especially exacerbated by the global health crisis. However, it involves a multidimensionality of factors connecting various variables in a spiral, with the performance of health establishments as the coordinating axis developing initiatives to combat malnutrition in the country.

## Conclusions

During the period 2010-2020 in Peru, the impact of public health expenditure on the reduction of malnutrition shows that in 10 departments, malnutrition was reduced; while in 14 departments, this indicator was not reduced. The most notable increases were in Pasco, where public health expenditure led to an increase in malnutrition by 17.61%, in Arequipa by 15.18%, in Lima by 9.85%, and in Cajamarca by 8.53%.

Public health expenditure has a negative relationship with malnutrition, while the unemployment rate shows a positive relationship with malnutrition, as being unemployed leads to a higher cause of malnutrition in the population due to lower income.

Malnutrition has a negative effect on public health expenditure, as little importance is given to public malnutrition policies, and public budgets are prioritized for other sectors.

Education has a negative effect on the rural population, mainly explained by the trend that as the human capital of the population increases, there is a migration from rural to urban areas, moving to central zones to improve their income levels.

Malnutrition has a positive effect on unemployment, as do education and inflation. High levels of malnutrition generate high unemployment rates, while the positive relationship between education and unemployment is explained by the excess accumulation of human capital exceeding job opportunities. The positive relationship between inflation and unemployment reflects the loss of purchasing power and delayed investment due to price fluctuations, leading to a decline in business confidence.

Exports have had a positive effect on GDP during the analyzed period, as higher exports result in a positive balance of payments, impacting foreign exchange and terms of trade, fostering GDP growth. Conversely, unemployment shows a negative relationship with GDP due to the still high unemployment rates in Peru, which negatively affect economic growth by reducing private investment and capital attraction. Inflation also shows a negative relationship with GDP, as the inflationary spiral leads to a loss of purchasing power, consequently affecting economic growth.

## Data Availability

Zenodo. Impact of public health expenditure on malnutrition among Peruvians during the period 2010-2020: A panel data analysis. 10.5281/zenodo.12736705 ( Castro et al., 2024). This project contains the following underlying data:
• Final results Panel data.xlsx This project contains the following extended data:
• Stata Commands.pdf •

Figure 1.Impact of Public Health Expenditure on Malnutrition by Department.png
•

Figure 2.Dynamics of the Identified Structural Equations in the Departments of Peru.jpg
•
Rectoral Resolution N° 760-2007_UCV_Code Of Ethics (1).pdf
•

STROBE_checklist_v4_combined.pdf Creative Commons Zero v1.0 Universal (CC0 License)
