# Supplementary material for: Impact of public health expenditure on malnutrition among Peruvians during the period 2010-2020: A panel data analysis
Source: F1000Res. 2025 Jan 17;13:990. Originally published 2024 Sep 2. [Version 3] doi: 10.12688/f1000research.153477.3 (PMC12008717; doi:10.12688/f1000research.153477.3)
Supplement: Peer review history

# Reviewer report: Reviewer response for version 3

**DOI:** 10.5256/f1000research.176735.r375314

**Published:** 2025-04-18

**Authors:** Anna Vipta Resti Mauludyani

**Affiliations:** 1 IPB University, Bogor, Indonesia

This paper is expected to provide a comprehensive point of view on the relationship between public health expenditure and malnutrition.

 However, the discussion still needs to be elaborated more, especially on the impact of the public health expenditure on malnutrition, which was also not found in the identified structural equations. Add also implications of the results, particularly more specific programs to address malnutrition problem.

Reviewer Expertise:

Community nutrition, nutrition policy analysis

I confirm that I have read this submission and believe that I have an appropriate level of expertise to confirm that it is of an acceptable scientific standard, however I have significant reservations, as outlined above.

# Reviewer report: Reviewer response for version 3

**DOI:** 10.5256/f1000research.176735.r377867

**Published:** 2025-04-18

**Authors:** Ratih Wirapuspita Wisnuwardani

**Affiliations:** 1 Department of Public Health Nutrition, Faculty of Public Health, Mulawarman University, Samarinda, East Kalimantan, Indonesia

Percy Junior Castro Mejía et al. conducted a study on the **Impact of public health expenditure on malnutrition among Peruvians during the period 2010-2020**, which can contribute to malnutrition prevention programs in Peru. This article will be very interesting for F1000 readers, as all countries are currently working to address nutrition issues.

 However, there are a few areas that need improvement, especially in data analysis and presentation of results.

 Tables 2-5: avoid using all tables. Using graphs would make it easier for readers to understand.

 Figure 2: Please simplify it, as it is very difficult to comprehend.

 The discussion is still not detailed enough and should compare with previous articles in meta-analysis and systematic reviews.

 The conclusion is too long; it should be shortened to a brief conclusion with recommendations.

Is the work clearly and accurately presented and does it cite the current literature?

Yes

If applicable, is the statistical analysis and its interpretation appropriate?

Yes

Are all the source data underlying the results available to ensure full reproducibility?

Yes

Is the study design appropriate and is the work technically sound?

Yes

Are the conclusions drawn adequately supported by the results?

Yes

Are sufficient details of methods and analysis provided to allow replication by others?

Yes

Reviewer Expertise:

Public health nutrition

I confirm that I have read this submission and believe that I have an appropriate level of expertise to confirm that it is of an acceptable scientific standard, however I have significant reservations, as outlined above.

# Reviewer report: Reviewer response for version 2

**DOI:** 10.5256/f1000research.173076.r339501

**Published:** 2024-12-09

**Authors:** Paraskevi Seferidi

**Affiliations:** 1 Imperial College London, London, UK

This paper explores associations between public health spending and malnutrition in Peru. There are some major concerns regarding the paper.

 Below are a few specific comments and recommendations:

- Overall paragraph structure needs to be improved. In the introduction, the authors are using small paragraphs of 1-2 sentences that provide unlinked information on various issues that are not connected. Try to restructure your introduction into 3-4 paragraphs, one per issue explored, e.g. one on the issue of hunger and undernutrition, one on the issue of inadequate health expenditure, and one on the association between health expenditure and malnutrition. I'd recommend skipping the literature review subheading and just summarise the existing evidence base pertaining to your research question into one comprehensive paragraph. Make sure that you only include relevant literature about associations of interest. For example, there is a lot of detail on specific papers that seem a bit irrelevant (e.g. Katoch, 2024). A lot of the literature cited is about associations with health and not malnutrition more specifically, but there is no clear distinction between the two (e.g. Edney, 2018). Description of several papers is unclear (e.g. Bernet 2018, Calva 2021) - writing needs to be improved to better communicate what these papers have found.
- The authors should provide a clear research gap. Currently they argue that there is plenty of literature looking at associations between public health spending and malnutrition (first paragraph of methods). So what is the rationale of conducting this work? Also, the authors have not provided a clear aim and/or objectives in their introduction.
- There is a lot of repetition in the methods, so a lot of work needs to be done to improve this.
- Methods seem to be misleading. Initially, the authors argue that the sample size is 2112 observations (not sure if this is households?), but then from the equations it looks like the level of observation is department. So, it looks like this is an ecological analysis with a sample size of 24. But then later on it seems that the authors may have done multi-level analysis, which also seems to be confirmed by the tables in the results. Overall, I find it impossible to assess the statistical analysis based on the text provided by the authors.
- I have concerns about the definition of malnutrition using a binary measure of being above or below a cut-off point for energy intake. The authors try to explain how they estimated this, but I’m afraid it is not clear what the purpose of the methodology by Herrera is and if it is appropriately used here. I would be cautious with naming this variable malnutrition, as this is probably the % of households that have (per capita??) energy intake below 2318 kcal. The interpretation of the results would be much different in this case.
- In the results, it looks like the authors explored associations by department, but it was not clear from the methods what was the methodological approach to estimate these.

Is the work clearly and accurately presented and does it cite the current literature?

No

If applicable, is the statistical analysis and its interpretation appropriate?

No

Are all the source data underlying the results available to ensure full reproducibility?

Yes

Is the study design appropriate and is the work technically sound?

No

Are the conclusions drawn adequately supported by the results?

No

Are sufficient details of methods and analysis provided to allow replication by others?

No

Reviewer Expertise:

public health nutrition

I confirm that I have read this submission and believe that I have an appropriate level of expertise to state that I do not consider it to be of an acceptable scientific standard, for reasons outlined above.

# Response

**Published:** 2025-02-04

**Authors:** Rogger Orlando Morán Santamaría

**Affiliations:** Universidad Cesar Vallejo, Trujillo, La Libertad, Peru

To

 Paraskevi Seferidi

 Imperial College London, London, UK

 Please accept greetings from the authors of the article.

 We appreciate your comments and know that they improve the quality of our finalised research.

 I am writing to inform you that the report has been uploaded with the suggested changes and that we will be able to continue with the process of publishing the article to the highest standards of quality.

 We hope we have met your requirements.

 We will await the new revision.

 Thank you very much.

# Reviewer report: Reviewer response for version 2

**DOI:** 10.5256/f1000research.173076.r335578

**Published:** 2024-10-31

**Authors:** Om Raj Katoch

**Affiliations:** 1 Department of Economics, Govt. Maulana Azad Memorial College Cluster University of Jammu, Jammu & Kashmir., Jammu & Kashmir, India

The authors have revised the paper based on the comments, and the quality has now been enhanced. In my opinion, it can be indexed now.

Is the work clearly and accurately presented and does it cite the current literature?

No

If applicable, is the statistical analysis and its interpretation appropriate?

Yes

Are all the source data underlying the results available to ensure full reproducibility?

Partly

Is the study design appropriate and is the work technically sound?

Partly

Are the conclusions drawn adequately supported by the results?

Partly

Are sufficient details of methods and analysis provided to allow replication by others?

Partly

Reviewer Expertise:

Nutrition

I confirm that I have read this submission and believe that I have an appropriate level of expertise to confirm that it is of an acceptable scientific standard.

# Reviewer report: Reviewer response for version 1

**DOI:** 10.5256/f1000research.168387.r321092

**Published:** 2024-09-09

**Authors:** Om Raj Katoch

**Affiliations:** 1 Department of Economics, Govt. Maulana Azad Memorial College Cluster University of Jammu, Jammu & Kashmir., Jammu & Kashmir, India

Review Report on the Research Paper: "Impact of Public Health Expenditure on Malnutrition Among Peruvians During the Period 2010-2020"

 I have reviewed your research paper and would like to provide feedback to help you enhance the quality and clarity of your work. Please consider the following points as you revise your manuscript:

**1. Background and Literature Review**

 The topic you address is highly relevant and significant in the field of public health. However, I recommend that you expand the literature review to include more recent studies. This will help you establish a stronger foundation for your research question and contextualize your findings within the current body of knowledge. Incorporating recent literature will also demonstrate the relevance and timeliness of your study. Cite these articles:

 Om Raj Katoch (2022 [Ref - 1]) https://doi.org/10.1016/j.nut.2021.111565

 Om Raj Katoch et al. (2024 [Ref - 2]) https://doi.org/10.1108/IJESM-10-2022-0007

 Om Raj Katoch (2024 [Ref - 3]) https://doi.org/10.1108/NFS-03-2023-0055

**2. Methodology**

 The methodology section requires further elaboration to improve both clarity and comprehensibility. While the current draft mentions the use of structural equations and regression analysis, it is essential to provide detailed explanations to support the robustness of your approach. Specifically, you should clearly specify the data sources used in your analysis, discussing their reliability and relevance to the study. Additionally, clarifying the sample size and justifying its adequacy for the conducted analysis is crucial to ensure the validity of your results. Moreover, a detailed description of the specific statistical techniques employed, along with an explanation of why these techniques were chosen, will help readers understand the analytical framework. It is also important to offer a clearer explanation of how control variables were selected and discuss their relevance to the model. Finally, addressing the assumptions made during the analysis and explaining how potential biases were mitigated will further enhance the methodological rigor of your paper. By incorporating these elements, you will provide a more comprehensive and transparent account of your research methodology.

**3. Policy Implications**

 The policy implications you discuss are crucial and well-founded. However, to make this section more impactful, consider providing more concrete recommendations for policymakers. For instance, elaborate on how increased investment in health services can be implemented effectively, particularly in rural areas. Additionally, consider discussing how these findings might be applied in other developing countries facing similar malnutrition challenges, thereby broadening the applicability of your research.

**4. Grammatical and Formatting Errors**

 The manuscript contains several grammatical errors and awkward phrasings that need to be addressed. A thorough proofreading is essential to correct these issues and ensure that your paper meets academic standards for publication and indexing. Additionally, pay attention to consistent formatting throughout the manuscript, including citation styles and headings

Is the work clearly and accurately presented and does it cite the current literature?

No

If applicable, is the statistical analysis and its interpretation appropriate?

Yes

Are all the source data underlying the results available to ensure full reproducibility?

Partly

Is the study design appropriate and is the work technically sound?

Partly

Are the conclusions drawn adequately supported by the results?

Partly

Are sufficient details of methods and analysis provided to allow replication by others?

Partly

Reviewer Expertise:

Nutrition

I confirm that I have read this submission and believe that I have an appropriate level of expertise to state that I do not consider it to be of an acceptable scientific standard, for reasons outlined above.

## References

[1] : Determinants of malnutrition among children: A systematic review. *Nutrition* .2022;96: 10.1016/j.nut.2021.111565 111565 10.1016/j.nut.2021.111565 35066367

[2] : Energy poverty and its impacts on health and education: a systematic review. *International Journal of Energy Sector Management* .2024;18(2) : 10.1108/IJESM-10-2022-0007 411-431 10.1108/IJESM-10-2022-0007

[3] : Tackling child malnutrition and food security: assessing progress, challenges, and policies in achieving SDG 2 in India. *Nutrition & Food Science* .2024; 10.1108/NFS-03-2023-0055 10.1108/NFS-03-2023-0055
